# Five million years in the darkness: A new troglomorphic species of *Cryptops* Leach, 1814 (Chilopoda, Scolopendromorpha) from Movile Cave, Romania

**DOI:** 10.3897/zookeys.1004.58537

**Published:** 2020-12-16

**Authors:** Varpu Vahtera, Pavel Stoev, Nesrine Akkari

**Affiliations:** 1 Zoological Museum, Biodiversity Unit, University of Turku, Turku, Finland University of Turku Turku Finland; 2 National Museum of Natural History and Pensoft Publishers, Sofia, Bulgaria National Museum of Natural History Sofia Bulgaria; 3 Naturhistorisches Museum Wien, Burgring 7, Wien 1010, Austria Naturhistorisches Museum Wien Vienna Austria

**Keywords:** Biospeleology, *Cryptops speleorex* sp. nov., Dobrogea, molecular phylogenetics, new species, troglomorphism

## Abstract

A new species of *Cryptops* Leach, 1814, *C. speleorex***sp. nov.**, is described from Movile Cave, Dobrogea, Romania. The cave is remarkable for its unique ecosystem entirely dependent on methane- and sulfur-oxidising bacteria. Until now, the cave was thought to be inhabited by the epigean species *C. anomalans*, which is widespread in Europe. Despite its resemblance to *C. anomalans*, the new species is well-defined morphologically and molecularly based on two mitochondrial (cytochrome *c* oxidase subunit I COI and 16S rDNA) and one nuclear (28S rDNA) markers. *Cryptops speleorex* sp. nov. shows a number of troglomorphic traits such as a generally large body and elongated appendages and spiracles, higher number of coxal pores and saw teeth on the tibia of the ultimate leg. With this record, the number of endemic species known from the Movile Cave reaches 35, which ranks it as one of the most species-rich caves in the world.

## Introduction

Located in the southeastern part of Romania not far from the Black Sea Coast, Movile Cave is the first known subterranean chemosynthesis-based ecosystem (Sarbu et al. 2019). Being completely isolated from the outside environment for 5.5 million years, the cave is remarkable for its unique ecosystem entirely dependent on methane- and sulfur-oxidising bacteria, which release nutrients through chemosynthesis for fungi and other cave animals along the food chain. This subterranean ecosystem is also notable for being rich in hydrogen sulfide, methane (1–2%), ammonia and CO_2_ (1.5–3.5%) whereas it is poor in O_2_ (7–16%). Relative humidity in the cave is 100% and there is no detectable air movement. The cave was first discovered in 1986 and since then, only a handful of people have visited it (Sarbu et al. 2019).

Despite its harsh living conditions, Movile Cave ecosystem is known to harbor a diverse and unique fauna. The cave hosts 51 invertebrate species, of which 34 species are endemic (Sarbu et al. 2019). Among these species, some present a number of unique adaptations to a troglobitic life in caves, such as the troglobiont water scorpion *Nepa anophthalma* Decu, Gruia, Keffer & Sarbu, 1994 (Hexapoda, Hemiptera, Nepidae); the nesticid and liocranid spiders *Kryptonesticus georgescuae* Nae, Serban & Weiss, 2018 (Araneae: Nesticidae) and *Agraecina cristiani* (Georgescu, 1989) (Araneae, Liocranidae); the cave leech *Haemopis caeca* Manoleli, Klemm & Sarbu, 1998 (Annelida, Hirudinea, Haemopidae) and the isopod *Armadillidium tabacarui* Gruia, Iavorschi & Sarbu, 1994 (Crustacea, Isopoda, Armadillidiidae) (Sarbu et al. 2019).

Five species of myriapods are hitherto discovered from the innermost parts of Movile viz. *Archiboreoiulus serbansarbui* Giurginca, Vănoaica, Šustr, & Tajovský, 2020 (Diplopoda), *Symphylella* Silvestri, 1902 sp. (Symphyla), *Geophilus alpinus* Meinert, 1870 and *Clinopodes carinthiacus* (Latzel, 1880) (Geophilomorpha) and a troglobitic population of *Cryptops anomalans* Newport, 1844 (Negrea 1993; Sarbu et al. 2019). It is worth mentioning that the latter taxon has been only studied morphologically (Negrea 1993, 2004).

Recently, we had the occasion to study freshly collected specimens of an undetermined species of the genus *Cryptops* Leach, 1814 from Movile Cave. Using both, morphological and molecular evidence, the cave specimens were compared with those of *C. anomalans* living on the surface, outside the cave. A phylogenetic analysis of 29 *Cryptops* specimens from different parts of Europe, including two from inside Movile Cave, based on two mitochondrial (cytochrome c oxidase subunit I COI and 16S rDNA) and one nuclear (28S rDNA) markers was performed. Morphological and molecular analyses confirmed that the cave specimens from Movile correspond to a new species, *Cryptops speleorex* sp. nov., that we describe herein. Additionally, we provide an annotated list and a key to the troglobitic *Cryptops* species in the world.

## Material and methods

All *Cryptops* specimens from Movile Cave were hand-collected by the biospeleologists Serban Sarbu and A. Hillebrand and preserved in 70% or 96% ethanol. Microphotographs were obtained with a Nikon DS-Ri-2 camera mounted on a Nikon SMZ25 stereomicroscope using NIS-Elements Microscope Imaging Software with an Extended Depth of Focus (EDF) patch. Images were edited in Photoshop CS6 and assembled in InDesign CS6. Material is shared between the **ISER** – Emil Racoviță Institute of Speleology, Bucharest, Romania; **IZB** – University of Belgrade – Institute of Zoology, Faculty of Biology, Belgrade, Serbia; **NHMW** – Naturhistorisches Museum Wien, Austria; **NMNHS** – National Museum of Natural History, Sofia, Bulgaria and the **ZMUT** – University of Turku – Zoological Museum, Finland. In addition to the type material of the new species we have morphologically studied material of *C. anomalans* from Serbia and Romania.

Morphological terminology follows Bonato et al. (2010).

Abbreviations: **T** – tergite, **S** – sternite.

### Molecular methods

Altogether 29 specimens from both inside and outside the Movile Cave were included in the phylogenetic analysis. Of these, 14 were sequenced in this study. Total DNA was extracted from the legs using NucleoSpinTissue kit (Macherey-Nagel) according to the standard protocol for human or animal and cultured cells. Samples were incubated overnight. One nuclear (28S rRNA) and two mitochondrial (cytochrome *c* oxidase subunit I, COI, and 16S rRNA) fragments were chosen for amplification since they have proven informative between closely related taxa (Vahtera et al. 2012, 2013). 28S rRNA fragment was amplified with the primers 28Sa/28Sb (Whiting et al. 1997), COI fragment with the primers LCO1490/HCO2198 (Folmer et al. 1994) and 16S rRNA with the primers 16Sa/16Sb (Xiong and Kocher 1991; Edgecombe et al. 2002). All primers had a universal tail (T7Promoter/T3) attached to them.

Polymerase chain reaction (PCR) amplifications were performed with MyTaqTM HS Red Mix. PCR was performed in a total volume of 23 μL containing 7.5 μL of MQ, 12.5 μL of MyTaq HS Red Mix, 2×, 0.5 μL of each primer (10 μM) and 2 μL of DNA template. PCR started with initial denaturation at 95 °C for 1 min and was followed by denaturation at 95 °C for 15 s. Annealing temperature for 28S rRNA and COI was 49 °C and 43 °C for 16S rRNA. Annealing lasted for 15 s and was followed by extension at 72 °C for 10 s. The last three steps were repeated 35 times. A negative control was included. PCR products were run in electrophoresis on 1% Agarose gel using Midori Green Advanced DNA Stain (Nippon Genetics). Samples were purified with an A’SAP PCR clean-up kit (ArcticZymes). Sequencing was performed by Macrogen Europe. The resulting chromatograms were visualized and assembled using the software Sequencher 5 (Gene codes corporation, USA). All new sequences are deposited in GenBank (See Table 1 for accession numbers).

**Table 1.** Specimens used in the molecular phylogeny and their GenBank accession numbers (specimens sequenced in this study in bold). Institutional abbreviations: ISER–Emil Racoviță Institute of Speleology, Bucharest, Romania; IZBU–University of Belgrade–Faculty of Biology, Institute of Zoology, Belgrade, Serbia; MCZ–Museum of Comparative Zoology, Harvard University; ZFMK–Museum Koenig, Bonn; ZSM–Bavarian State Collection of Zoology, Munich; ZMUT–Zoological Museum, University of Turku, Finland.

Species	Lab code	Voucher ID number	Voucher	Country	COI	16S	28S
*Cryptops speleorex* sp. nov.	K3	http://mus.utu.fi/ZMUT.MYR-TYPE001	ZMUT	Romania	**MW240507**	**MW243978**	**MW243648**
*C. speleorex* sp. nov.	K4		ISER	Romania	**MW240508**	**MW243977**	**MW243649**
*C. anomalans*	1a		IZB	Serbia	**MW240504**	**MW243967**	**MW243651**
*C. anomalans*	1b		IZB	Serbia	**MW240505**	**MW243968**	**MW243652**
*C. anomalans*	2		IZB	Serbia	**MW240511**	–	MW243642
*C. anomalans*	3		IZB	Serbia	**MW240515**	MW243970	MW243643
*C. anomalans*	4		IZB	Serbia	MW240503	MW243979	MW243654
*C. anomalans*	7		IZB	Serbia	MW240506	MW243969	MW243653
*C. anomalans*	8		IZB	Serbia	**MW240512**	MW243971	MW243644
*C. anomalans*	9		IZB	Serbia	**MW240514**	**MW243973**	MW243645
*C. anomalans*	12		IZB	Serbia	**MW240516**	**MW243974**	**MW243646**
*C. anomalans*	13		IZB	Serbia	**MW240513**	**MW243972**	**MW243647**
*C. anomalans*	54a		ISER	Romania	**MW240510**	MW243975	MW243650
*C. anomalans*	57a		ISER	Romania	**MW240509**	MW243976	MW243641
*C. anomalans*		ZFMK-MYR 1048	ZFMK	Germany	KM491639	–	–
*C. anomalans*		ZFMK-MYR 1047	ZFMK	Germany	KM491699	–	–
*C. anomalans*		ZFMK-MYR 1379	ZFMK	Germany	KM491703	–	–
*C. anomalans*		ZFMK-MYR 4072	ZFMK	Germany	KM491706	–	–
*C. anomalans*		ZSM-ART-JSP130812-004	ZSM	Germany	KU497151	–	–
*C. anomalans*		ZSM-ART-JSP110624-001	ZSM	Germany	KU497158	–	–
*C. anomalans*		ZSM-ART-JSP141105-017	ZSM	Germany	KU497159	–	–
*C. anomalans*		IZ-131458	MCZ	UK	KF676499	KF676457	KF676353
*Cryptops* sp.		ZFMK-MYR-1185	ZFMK	Austria	KM491620	–	–
*Cryptops* sp.		ZFMK-MYR 3662	ZFMK	Germany	KU342042	–	–
*Cryptops* sp.		ZSM-ART-JSP150118-047	ZSM	Slovenia	KU497143	–	–
*Cryptops* sp.		ZSM-ART-JSP110425-008	ZSM	Croatia	KU497153	–	–
*C. croaticus*		ZFMK-MYR 3320	ZFMK	Austria	KU342049	–	–
*C. hortensis*		IZ-130582	MCZ	UK	JX422662	JX422684	JX422582
*C. parisi*		IZ-130592	MCZ	UK	KF676502	KF676460	KF676356
*Scolopendra cingulata*		IZ-131446	MCZ	Spain	HM453310	HM453220	AF000782

### Phylogenetic analyses

Most specimens included in the analysis had all three markers successfully sequenced. To obtain more geographic variation in the dataset, 15 *Cryptops* specimens (mostly from Wesener et al. 2016) from GenBank (Table 1) were additionally included in the phylogenetic analysis. Of these, 12 had only COI available. Multiple sequence alignments were performed in MAFFT7 online service (Katoh et al. 2019; Kuraku et al. 2013). Sequences were trimmed in Mesquite v 3.10 (Maddison and Maddison 2019) after which the three separate data sets were concatenated with SequenceMatrix (Vaidya et al. 2011) for the phylogenetic analyses. The final molecular matrix including all three data sets (COI, 16S, 28S) consisted of 1561 characters and 29 taxa (excluding outgroup).

Phylogenetic analysis was conducted using both parsimony and maximum likelihood as optimality criteria. Parsimony analysis was done with TNT v. 1.5 (Goloboff and Catalano 2016) treating gaps as missing data. The search strategy consisted of 100 replications, and of 10 rounds of both ratchet and tree drifting followed by tree fusing (Goloboff 1999). Command xmult was executed until 50 independent hits of the shortest tree were found. A strict consensus of the most-parsimonious trees was produced. The command ‘blength’ was used to report the branch lengths of the resulting trees. Jackknife (Farris et al. 1996) resampling method with 1000 replicates and with a probability of a character removal being 0.36 was applied to estimate nodal support. Maximum likelihood analysis of the combined data was conducted RAxML v. 8 (Stamatakis 2014) in the CIPRES portal (Miller et al. 2010). The three genes were separated into different partitions. Unique general time-reversible (GTR) model of sequence evolution (RAxML implements only GTR-based models of nucleotide substitutions) with corrections for a discrete gamma distribution (GTR+ Γ) was used. Nodal support values were estimated using the rapid bootstrap algorithm with 1000 replicates together with GTR-CAT model (Stamatakis et al. 2008). The mitochondrial genes (16S+COI) and the nuclear ribosomal 28S were additionally analysed separately using the same search strategy as was used for the combined data.

Uncorrected p-distances of aligned COI, 16S and 18S data were calculated with MEGA v. 7.0.21 (Kumar et al. 2016).

## Results

**Order Scolopendromorpha Pocock, 1895**

**Family Cryptopidae Kohlrausch, 1881**

### Genus *Cryptops* Leach, 1814

#### Cryptops (Cryptops) anomalans

Taxon classificationAnimaliaScolopendromorphaCryptopidae

Newport, 1844

B79D1FC1-76EE-50BB-8AA6-4933964B0D8F

##### Material examined.

**Romania**: SE Romania: Lalomiţa County, Călugărească Forest, 18.II.2016, leg. and det. S. Baba, 1 subad. ex. (ISER); Lalomiţa County, Călugărească Forest, oak forest, 28.II.2019, leg. and det. S. Baba, 2 ex. (ISER) (lab code 54a); Lalomiţa County, Călugărească Forest, rotten wood, 13.III.2016, leg. and det. S. Baba, 1 ex. (ISER); Bucharest, Herăstrău Park, under stones, 10.X.2019, leg. and det. S. Baba, 1 ex. (ISER) (lab code 57a); Mangalia, Obanul Mare, Cave Drilling, -3 m, 10.VIII.1999, det. St. and A. Negrea, 1 ex. (ISER); Mangalia, Obanul Mare, Cave Drilling, -8 m, 27.V.2000, det. St. and A. Negrea, 1 ex. (ISER); Mangalia, Obanul Mare, Cave Drilling, -8 m, 28.VI.2000, det. St. and A. Negrea, 1 ex. (ISER); Mangalia, Obanul Mare, Cave Drilling, -12 m, 27.V.2000, det. St. and A. Negrea, 1 ex. (ISER). **Serbia**: Valley of the Izbice River, v. Izbice, near Novi Pazar, SW Serbia (43°07.333'N, 20°34.354'E; elevation about 700 m a.s.l.): 5♂, 5♀, collected in 2012 (May-October), leg. D. Stojanović (lab code 1) (IZB); Prolom Banja Spa, near Kuršumlija, southern Serbia (43°02.449'N, 21°23.448'E; elevation about 620 m a.s.l.): 3♀, collected 30.04.2016., leg. D. Stojanović (lab code 2) (IZB); village Kacabać, near Bojnik, Leskovac, southern Serbia (43°03.415'N, 21°46.368'E; elevation about 200 m a.s.l.): 2♂, 1♀, collected 01.05.2016., leg. D. Stojanović (lab code 3) (IZB); Pećina Rasnica 1 Cave, village Rasnica, near Pirot, SE Serbia: 1♂, 1♀, collected 18.07.2018., leg. D. Antić (lab code 4) (IZB); Novopazarska Banja Spa, near Novi Pazar, SW Serbia (43°09.269'N, 20°33.132'E; elevation about 650 m a.s.l.): 3♂, 4♀, collected 30.05.2012., leg. D. Stojanović (IZB); Spomen Park, Leskovac, southern Serbia (42°59.051'N, 21°56.349'E; elevation about 200 m a.s.l.): 1♀, collected 28.07.2012., leg. D. Stojanović (IZB); pine forest near the Đurđevi Stupovi Monastery, Novi Pazar, SW Serbia (43°09.183'N, 20°30.049'E): 1♀, collected 15.05.2015., leg. D. Stojanović (lab code 7) (IZB); village Dobanovci, near Surčin, Belgrade, Serbia (44°49.197'N, 20°13.334'E): 1♀, collected 03.11.2013., leg. D. Stojanović (lab code 8) (IZB); Bojčinska šuma forest, village Progar-Jakovo, near Surčin, Belgrade, Serbia (44°43.528'N, 20°09.245'E): 3♀, 1♂, collected 09.06.2013., leg. D. Stojanović, K. Bjelanović (lab code 9) (IZB); Vrla River, Mt. Vlasina, near “Rosa” water factory, v. Topli Do, near Surdulica, SE Serbia (42°38.213'N, 22°17.565'E; elevation about 1070 m a.s.l.): 1♂, collected 07.07.2011, leg. D. Stojanović (IZB); Višnjička Banja, Belgrade, Serbia (44°49.073'N, 20°32.337'E; elevation about 350 m a.s.l.): 1♂, collected 09.06.2006, leg. Ž. Pavković (IZB); Spomen Park, Leskovac, southern Serbia (42°59.051'N, 21°56.349'E; elevation about 200 m a.s.l.): 3♀, 1♂, collected 14.04.2012., leg. D. Stojanović (lab code 12) (IZB). **Bulgaria**: Pirin Mts, between Sandanski and Lilyanovo, 12.8.1988, litter, mainly *Platanus*, P. Beron leg. 1 ex. (NMNHS) (Figs 2B, 3B, 4B,D, 5B).

#### Cryptops (Cryptops) speleorex sp. nov.

Taxon classificationAnimaliaScolopendromorphaCryptopidae

2DB43C46-D6D3-5F10-AEA7-8CAAE5D82666

http://zoobank.org/8A28E7DF-168B-485C-8A0C-CD7EB218E650

**Figure 1.**
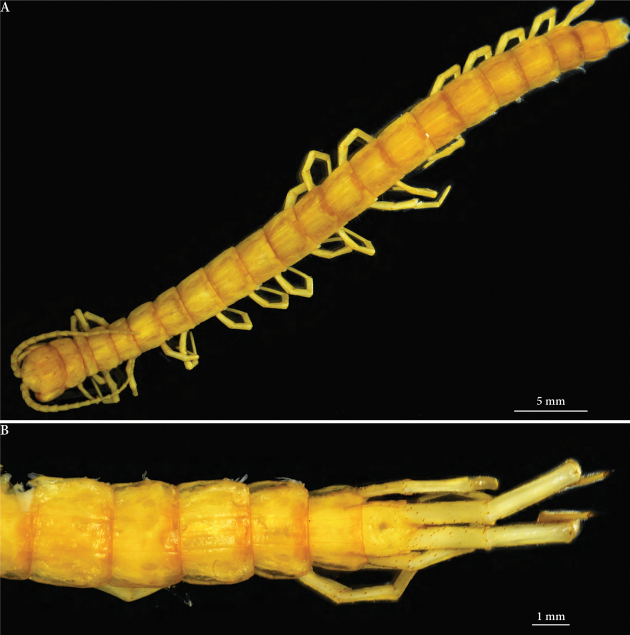
*Cryptops speleorex* sp. nov. **A** holotype, habitus, dorsal view **B** paratype (ZMUT), posteriormost segments and ultimate legs, dorsal view.

**Figure 2.**
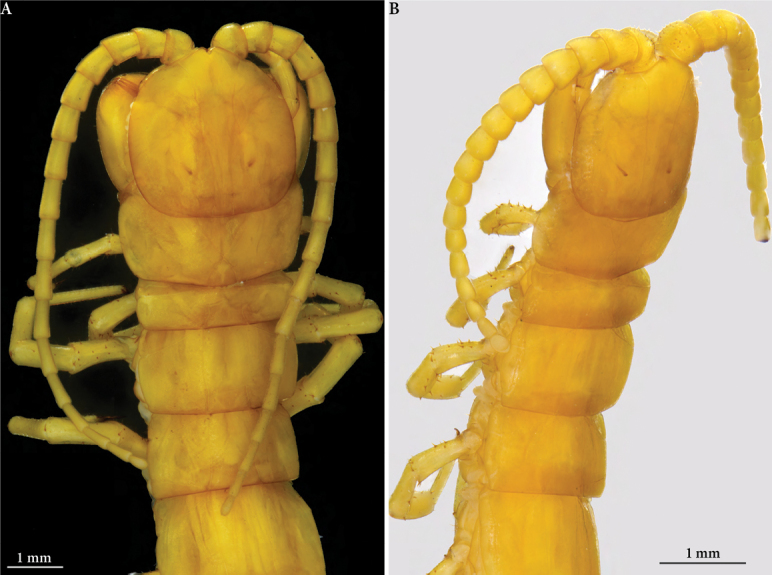
*Cryptops* spp., head and anteriormost segments **A***Cryptops speleorex* sp. nov., holotype, dorsal view **B***Cryptops anomalans*, Pirin Mts (Bulgaria), dorsolateral view (slightly apical).

**Figure 3.**
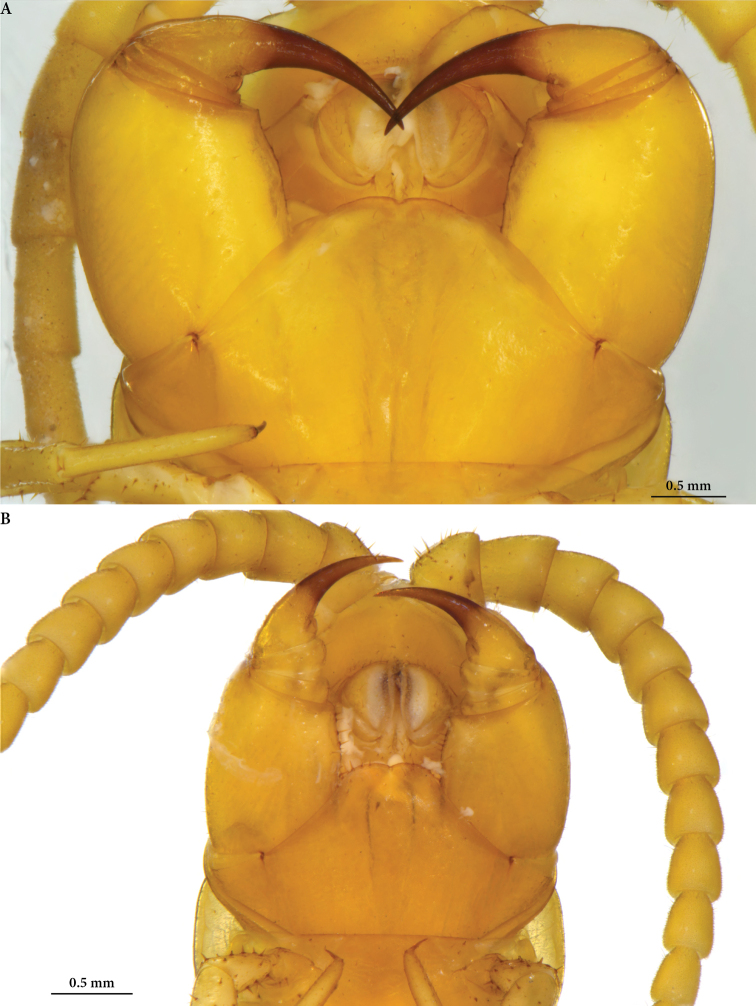
*Cryptops* spp., forcipular coxosternum, ventral view **A***Cryptops speleorex* sp. nov., holotype **B***Cryptops anomalans*, Pirin Mts (Bulgaria).

**Figure 4.**
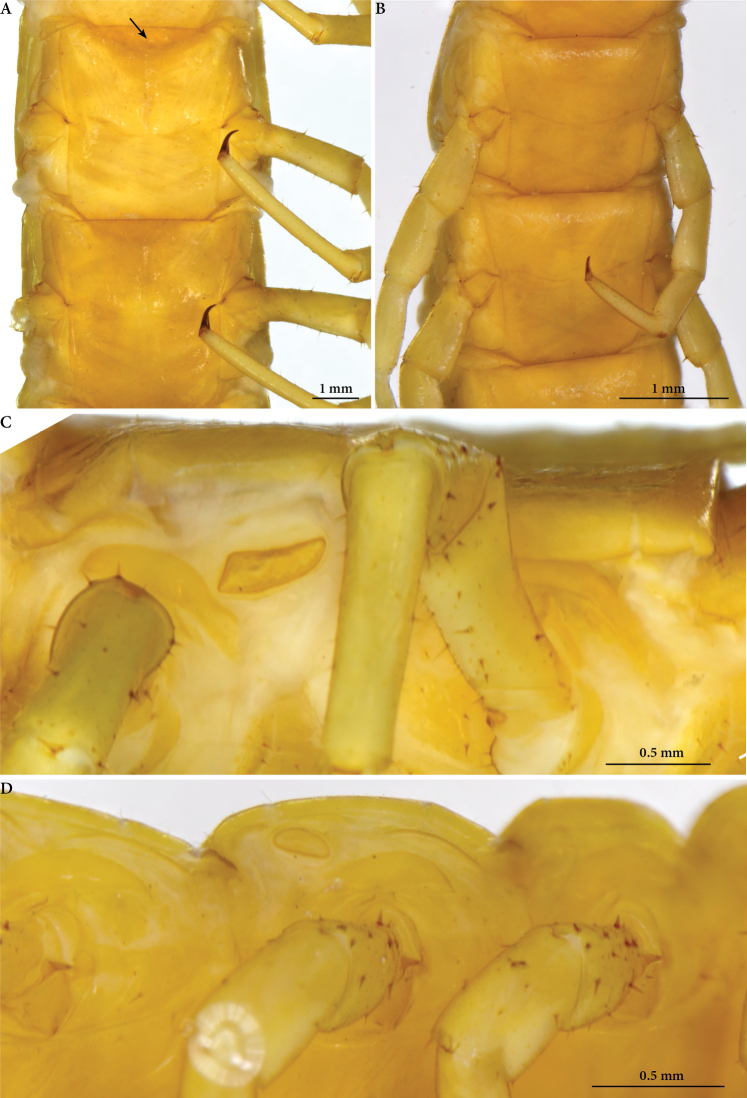
*Cryptops* spp., sternites 8–9 and spiracles **A, B** sternites 8–9 **A***Cryptops speleorex* sp. nov., holotype, arrow indicating the endosternite **B***Cryptops anomalans*, Pirin Mts (Bulgaria) **C, D** spiracles **C***Cryptops speleorex* sp. nov., holotype **D***Cryptops anomalans*, Pirin Mts (Bulgaria).

**Figure 5.**
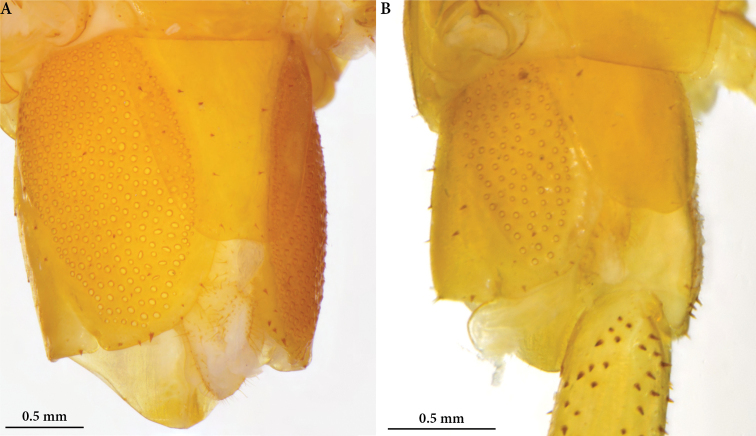
*Cryptops* spp., Coxopleural pore field **A***Cryptops speleorex* sp. nov., holotype **B***Cryptops anomalans*, Pirin Mts (Bulgaria).

**Figure 6.**
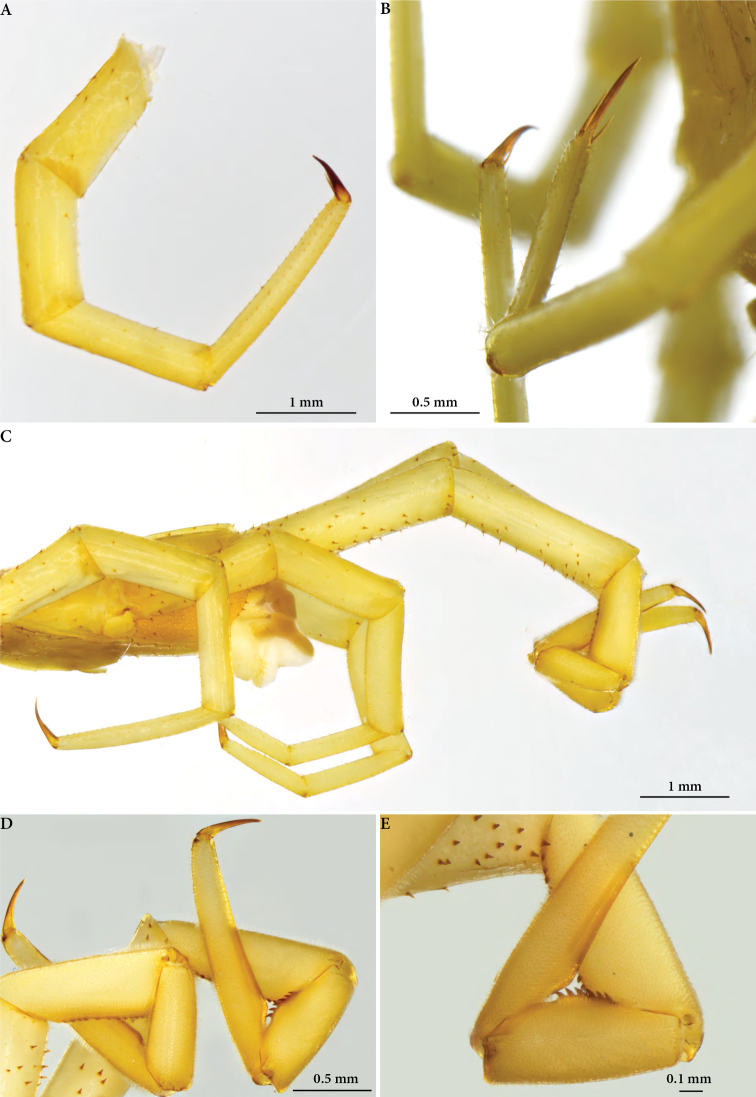
*Cryptops speleorex* sp. nov., legs. **A** holotype, walking leg **B** paratype (ZMUT), walking leg, close-up of apical claw **C** holotype, ultimate legs, lateral view **D, E** paratype (NHMW), distal articles of ultimate teeth showing saw teeth.

Figs 1A, B , 2A , 3A , 4A, C , 5A , 6A–C

##### Previous records.

*Cryptops anomalans*: Negrea, 1993: p. 87 and all subsequent records (Negrea 1994, 1997, 2004; Negrea and Minelli 1994; Sarbu et al. 2019).

##### Material examined.

***Holotype***: **Romania**: Constanța County, Mangalia, Movile Cave (Peștera Movile), Lake Hall, June, 2014, leg. S. Sarbu, 1 ex. (NMNHS, Myriapoda Collection Id: 10 812); ***Paratypes***: same locality and collector leg. S. Serban, 1 ex. (NHMW10177); same locality, 22.XI.2017, leg. A. Hillebrand, 1 ad. ex., identified as *C. anomalans* by Stefan Baba (ISER); 1 ad. ex., same locality, date and collector, identified as *C. anomalans* by Stefan Baba (http://mus.utu.fi/ZMUT.MYR-TYPE001).

##### Diagnosis.

A species morphologically similar to *Cryptops anomalans*, but differing from it by the much elongated antennae and legs, generally less setose forcipules and body, coxopleures with more than 300 coxal pores (vs. less than 100 in *anomalans*), ultimate leg with 13–17 saw teeth on tibia (usually 7–10, occasionally 12 in *anomalans*), and larger and elongated spiracles (see Table 2). Genetically, *Cryptops speleorex* sp. nov. differs from the *C. anomalans* specimens from Romania and Serbia by 9.2–12.2% in COI and 6.6–8.7% in 16S rDNA.

**Table 2.** Differences in morphological characters between *Cryptops anomalans* and *C. speleorex* sp. nov.

Morphological character	*Cryptops anomalans*	*Cryptops speleorex* sp. nov.
Body size (mm)	25–50	>46–52
Antennae length	Until posterior end of T3	Until mid of T5
Antennal article 7 L/W (mm)	0.5 × 0.25	1.0 × 0.5
Antennae: spines on basal articles	Present, numerous	Lacking or just a few
Ultimate leg length	7.65 mm	13.25 mm
Ultimate leg pretarsus (mm)	0.25	1
Ultimate leg saw teeth on tibia and tarsus 1	Tibia: 7–12 (usually 7–10); Tarsus: 3–5	Tibia: 13–17; Tarsus: 5–6
Legs	Short, compact, pretarsus short	Strongly elongated, pretarsus long
Spiracles	Ovoid, small to medium sized (Fig. 4D)	Strongly elongated, large (Fig. 4C)
Forcipular trochanteroprefemur	With spines medially (4–6)	Without spines, just stout setae
Coxopleural pore field	Approx. 2/3 of coxapleura; composed of less than 100 pores (86–90)	Approx. 4/5 of coxopleura; composed of more than 310 pores (317–320)

##### Description (holotype).

Length (anterior margin of head plate to posterior margin of telson) approx. 52 mm (46 mm in an adult paratype) (Figs 1A, B). Head plate (Fig. 2A) 3.2 mm long, 3.4 mm broad; antenna approx. 10 mm long. Body yellow-brownish (Fig. 1A); antennae and legs pale yellow; posterior edge of head and tergites with irregular light brownish band, darker in the middle (Figs 1B, 2A); forcipular tarsungulum and leg claws dark brown. Head plate overlaps approx. 1/3 of tergite 1; head plate slightly broader than long (3.2 mm × 3.4 mm), posterior corners strongly rounded, sides convex outwards, anterior apex slightly indented at the base of antennae, bisected by longitudinal median furrow; paramedian sutures diverging anteriorly on head plate; head punctate, sparsely covered with fine setae.

***Antenna*** relatively long, extending to the middle of tergite 5 when folded backward (Figs 1A, 2A); composed of 17 articles; article length formula: 17<1<2=16<3=4=13=14<5=6=11=12<7–10; basal two articles relatively stout, in general articles increase in length to a maximum at articles 7–10, then gradually shortening; article 17 is more than half length of article 16 (approx. 60%); articles 5–10 much longer than wide, length up to 3 times the width. All surfaces of antennal articles with scattered long setae, densest on articles 1–3; short, fine setae abundant on all articles except for articles 1 and 2, as well as basal part of 3.

***Clypeus*** with 2 setae; prelabral setae in one row of 21–22; 4 short setae between clypeus and prelabral row, irregularly or more evenly scattered. Labral mid piece with a short, but well-developed tooth; side pieces rounded (Fig. 3A).

***Forcipular segment*** anterior margin of coxosternite convex on each side, with a weak median diastema, fringed by 2 marginal setae on each side. Surface of coxosternite (Fig. 3A) covered with scarce short setae, 10–15 in total; trochanteroprefemur stout, median margin slightly expanded proximally, with 4 setae; femur and tibia very short; tarsungulum long, curved, almost equal in length to trochanteroprefemur’s height.

***Maxilla 2*** with a well-developed pretarsus; dorsal brush white, dense, situated on the distalmost part of article 3 of telopodite. Proximal side of first maxillary telopodite covered by 10–15 setae (Fig. 3A).

***Tergites*** Tergite 1 with a complete anterior transverse suture and cruciform sutures (Figs 1A, 2A). Oblique sutures present on tergites 2–8; complete paramedian sutures on tergites 2–20; lateral crescentic sulci visible on tergites 6–20; all tergites nearly devoid of setae, occasionally individual scattered short setae. Tergite 21 longer than wide, posterior margin subtriangular, with rounded apex; shallow median depression along posterior half of tergite (Fig. 1B).

***Sternites*** 1–2 and 19–21 without transverse and median sutures; S 3–18 with median longitudinal and curved transverse sutures, more prominent from sternite 5 onward (Fig. 4A). All sternites covered by minute setae. Endosternite: subtrapezoidal, lateral margins very slightly convex, posterior margin slightly concave in the middle; surface with several (6–10) moderately long and sparse setae.

***Spiracles*** strongly elongated on T3, reducing in size towards the posterior end of the body; slit-like (Fig. 4C).

***Coxopleural pore field*** elliptical, covering 4/5 of surface, with more than 310 coxal pores (317–320), extending nearly to posterior margin of coxopleuron (Fig. 5A). Approx. 15–20 sparse spiniform setae emerging between pores and from the dorsal and posterior margins of coxopleuron.

***Legs*** generally long; leg 10: prefemur 1.47 mm long, femur 1.59 mm, tibia 1.76 mm, tarsus 2.35 mm, pretarsus 0.7 mm. All tarsi single (Fig. 6A). Walking legs (Fig. 6A, B) smooth, generally poor in setae; spiniform setae sparsely present on the surface of prefemur, and occasionally also on the femur; all pretarsi long, with an anterior and posterior accessory spines of different size, the larger being 2/3^rd^ of pretarsus; accessory spines absent on leg 21; 20 leg: prefemur, femur and tibia slightly swollen; femur and tibia being slightly concave at midlength; a specific field of dense, minute setae present on the ventral, lateral and mesal sides of prefemur, femur and part of tibia.

***Ultimate leg*** (Fig. 6C): prefemur 3.61 mm long, femur 3.05 mm, tibia 1.94 mm, tarsus 1: 1.28 mm, tarsus 2: 2.22, pretarsus 0.56 mm.; numerous robust spiniform setae on the ventral, mesal and less so on lateral and dorsal sides of prefemur; spiniform setae present also on the ventral and mesal sides of femur; tibia, tarsus 1 and tarsus 2 covered by tiny dense setae on all sides; 13–14 saw teeth on tibia (17 in an adult paratype) and 5–6 on tarsus 1.

##### Etymology.

The species epithet is a noun in apposition, meaning "king of the cave", referring to the species top position in the food chain of the Movile ecosystem.

##### Distribution.

The species is hitherto known only from the aphotic zone of the Cave Movile in the southern part of Romanian Dobrogea.

##### Ecological remarks.

*Cryptops speleorex* sp. nov. is the largest invertebrate species in Movile Cave. It has been observed feeding on terrestrial isopods (*Trachelipus troglobius* Tabacaru & Boghean, 1989, *Armadillidium tabacarui* Gruia, Iavorschi & Sarbu, 1994), smaller beetles, Diplura or spiders (Sarbu et al. 2019).

## Phylogenetic analyses

**Figure 7.**
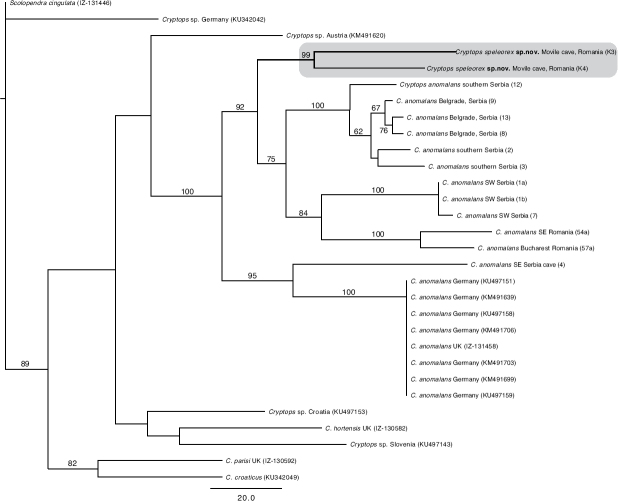
The single most parsimonious tree of length 1586 steps with jackknife resampling values > 50% shown on the nodes. Branch lengths represent the number of optimized character-state changes.

Parsimony analysis resulted in a single most-parsimonious (MP) tree of length 1586 steps (Fig. 7). Two *C. speleorex* sp. nov. specimens collected from Movile Cave (samples K3 and K4) group within *C. anomalans* as a separate clade supported by jackknife resampling value (hereafter JF) of 99. The phylogeny shows the Movile Cave clade being evolutionary most closely related to the clade (JF = 75) including *C. anomalans* samples from southern Serbia and Belgrade area (JF = 100) and Romania and SW Serbia (JF = 84). This Serbian/Romanian clade forms a sister group with the clade (JF = 95) containing a single *C. anomalans* specimen (lab code 4) from southeast Serbia (collected from a cave) and identical sequences of *C. anomalans* from London, UK and different parts of Germany (JF = 100). All specimens above form a clade with strong support (JF = 92). Outside this clade are *Cryptops* sp. from Austria and an unsupported clade containing *Cryptops* spp. from Croatia and Slovenia together with *C. hortensis* (Donovan, 1810). Basal to these are resolved *C. parisi* Brolemann, 1920 and *C. croaticus* Verhoeff, 1931 (JF = 82) followed by *Cryptops* sp. from Germany.

**Figure 8.**
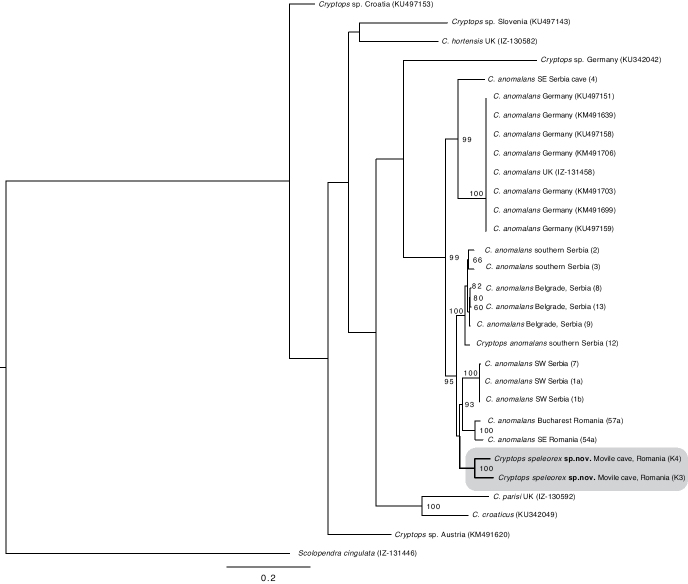
Likelihood tree with bootstrap values > 50% shown for each node.

Regarding the placement of *C. speleorex* sp. nov. and the relationships among the *C. anomalans* specimens, the likelihood analysis (Fig. 8) resulted in a mostly congruent tree topology with the parsimony tree, the only difference being that in the parsimony analysis *C. speleorex* sp. nov. is resolved basal to the Serbian/Romanian clade whereas in the likelihood tree it is resolved within it. The *C. speleorex* sp. nov. specimens form a clade supported by bootstrap value (hereafter BS) of 100. *Cryptops speleorex* sp. nov. groups together with the *C. anomalans* specimens from Serbia (excluding a single Serbian *C. anomalans* specimen, lab code 4) and Romania. All the specimens above form a sister clade to a group including *C. anomalans* specimens from Serbia (lab code 4), Germany and the UK. As in the parsimony analysis, the additional *Cryptops* species (other than *C. anomalans*) were resolved as basal to *C. anomalans*. Their internal grouping varies from that in the parsimony tree, which is not surprising due to the lack of nodal support in the basal-most nodes.

When analyzed separately (only likelihood, tree not shown), the mitochondrial COI and 16S resolved *C. speleorex* sp. nov. as a distinct clade (BS = 100) within *C. anomalans* specimens, the tree topology regarding *C. speleorex* sp. nov./*C. anomalans* being identical to that of the parsimony tree. Not surprisingly, the level of variation in the nuclear 28S was low and the likelihood analysis based on it could not resolve the relationships among the *C. anomalans/C.speleorex* sp. nov. specimens (tree not shown).

### Pairwise distances

Pairwise distances between the samples by each marker are shown in Tables 3–5. The differences between *C. speleorex* sp. nov. and the closest clade (Fig. 7) comprising of *C. anomalans* specimens from Romania and Serbia are 9.2–12.2% (COI) and 6.6–8.7% (16S rDNA). Nuclear 28S rDNA was conservative and showed almost no variation (0–0.3%) between these specimens. The difference between the new species and the rest of the *C. anomalans* specimens (Serbia (lab code 4), Germany and UK) is 13.8–15.5% (COI). In respect to 16S the differences were 10.7–12.5% and 9.9–11.2% between the new species and the Serbian (lab code 4) and *C. anomalans* from London, UK, respectively. Intraspecific difference between the two *C. speleorex* sp. nov. specimens is 8.5% in COI and 6.6% in 16S.

**Table 3.** Estimates of evolutionary divergence between sequences. COI: The number of base differences per site from between sequences are shown. The analysis involved 30 nucleotide sequences. Codon positions included were 1^st^+2^nd^+3^rd^+Noncoding.

All positions containing gaps and missing data were eliminated. There were a total of 556 positions in the final dataset.																														
		1	2	3	4	5	6	7	8	9	10	11	12	13	14	15	16	17	18	19	20	21	22	23	24	25	26	27	28	29
**1**	*Scolopendra cingulata* (IZ-131446)																													
**2**	*Cryptops anomalans* UK (IZ-131458)	0.248																												
**3**	*C. anomalans* Germany (KM491639)	0.248	0.000																											
**4**	*C. anomalans* Germany (KM491699)	0.248	0.000	0.000																										
**5**	*C. anomalans* Germany (KM491703)	0.248	0.000	0.000	0.000																									
**6**	*C. anomalans* Germany (KM491706)	0.248	0.000	0.000	0.000	0.000																								
**7**	*C. anomalans* Germany (KU497151)	0.248	0.000	0.000	0.000	0.000	0.000																							
**8**	*C. anomalans* Germany (KU497158)	0.248	0.000	0.000	0.000	0.000	0.000	0.000																						
**9**	*C. anomalans* Germany (KU497159)	0.248	0.000	0.000	0.000	0.000	0.000	0.000	0.000																					
**10**	*C. anomalans* SE Serbia cave (4)	0.237	0.110	0.110	0.110	0.110	0.110	0.110	0.110	0.110																				
**11**	*C. anomalans* SW Serbia (1a)	0.223	0.137	0.137	0.137	0.137	0.137	0.137	0.137	0.137	0.144																			
**12**	*C. anomalans* SW Serbia (1b)	0.223	0.137	0.137	0.137	0.137	0.137	0.137	0.137	0.137	0.144	0.000																		
**13**	*C. anomalans* SW Serbia (7)	0.225	0.138	0.138	0.138	0.138	0.138	0.138	0.138	0.138	0.149	0.005	0.005																	
**14**	*Cryptops speleorex* sp. nov. Movile cave, Romania (K3)	0.246	0.155	0.155	0.155	0.155	0.155	0.155	0.155	0.155	0.153	0.121	0.121	0.126																
**15**	*Cryptops speleorex* sp. nov. Movile cave, Romania (K4)	0.225	0.138	0.138	0.138	0.138	0.138	0.138	0.138	0.138	0.142	0.095	0.095	0.101	0.085															
**16**	*C. anomalans* Bucharest Romania (57a)	0.212	0.138	0.138	0.138	0.138	0.138	0.138	0.138	0.138	0.135	0.074	0.074	0.079	0.112	0.103														
**17**	*C. anomalans* SE Romania (54a)	0.225	0.146	0.146	0.146	0.146	0.146	0.146	0.146	0.146	0.142	0.092	0.092	0.097	0.122	0.117	0.040													
**18**	*C. anomalans* southern Serbia (2)	0.243	0.129	0.129	0.129	0.129	0.129	0.129	0.129	0.129	0.138	0.103	0.103	0.108	0.104	0.097	0.088	0.099												
**19**	*C. anomalans* Belgrade, Serbia (8)	0.239	0.133	0.133	0.133	0.133	0.133	0.133	0.133	0.133	0.140	0.097	0.097	0.103	0.110	0.094	0.086	0.094	0.023											
**20**	*C. anomalans* Belgrade, Serbia (13)	0.239	0.131	0.131	0.131	0.131	0.131	0.131	0.131	0.131	0.138	0.095	0.095	0.101	0.108	0.092	0.085	0.092	0.022	0.002										
**21**	*C. anomalans* Belgrade, Serbia (9)	0.239	0.131	0.131	0.131	0.131	0.131	0.131	0.131	0.131	0.138	0.095	0.095	0.101	0.108	0.092	0.085	0.092	0.022	0.002	0.000									
**22**	*C. anomalans* southern Serbia (3)	0.239	0.131	0.131	0.131	0.131	0.131	0.131	0.131	0.131	0.138	0.104	0.104	0.110	0.112	0.092	0.099	0.106	0.032	0.027	0.025	0.025								
**23**	*C. anomalans* southern Serbia (12)	0.241	0.137	0.137	0.137	0.137	0.137	0.137	0.137	0.137	0.135	0.094	0.094	0.099	0.106	0.090	0.090	0.101	0.040	0.029	0.027	0.027	0.043							
**24**	*C. hortensis* UK (IZ-130582)	0.243	0.203	0.203	0.203	0.203	0.203	0.203	0.203	0.203	0.182	0.174	0.174	0.178	0.173	0.189	0.180	0.187	0.198	0.200	0.198	0.198	0.191	0.191						
**25**	*Cryptops* sp. Austria (KM491620)	0.228	0.178	0.178	0.178	0.178	0.178	0.178	0.178	0.178	0.167	0.182	0.182	0.185	0.174	0.167	0.176	0.183	0.180	0.173	0.173	0.173	0.185	0.182	0.169					
**26**	*Cryptops* sp. Croatia (KU497153)	0.230	0.182	0.182	0.182	0.182	0.182	0.182	0.182	0.182	0.180	0.200	0.200	0.203	0.201	0.191	0.203	0.209	0.198	0.196	0.194	0.194	0.194	0.192	0.156	0.156				
**27**	*C. parisi* UK (IZ-130592)	0.221	0.192	0.192	0.192	0.192	0.192	0.192	0.192	0.192	0.173	0.176	0.176	0.182	0.191	0.182	0.171	0.180	0.171	0.169	0.167	0.167	0.167	0.171	0.196	0.192	0.185			
**28**	*C. croaticus* (KU342049)	0.239	0.201	0.201	0.201	0.201	0.201	0.201	0.201	0.201	0.185	0.173	0.173	0.176	0.192	0.178	0.174	0.180	0.194	0.194	0.192	0.192	0.196	0.196	0.169	0.192	0.192	0.137		
**29**	*Cryptops* sp. Slovenia (KU497143)	0.255	0.192	0.192	0.192	0.192	0.192	0.192	0.192	0.192	0.185	0.189	0.189	0.192	0.201	0.203	0.189	0.203	0.201	0.205	0.207	0.207	0.201	0.196	0.173	0.207	0.180	0.187	0.165	
**30**	*Cryptops* sp. Germany (KU342042)	0.223	0.201	0.201	0.201	0.201	0.201	0.201	0.201	0.201	0.187	0.192	0.192	0.196	0.210	0.194	0.178	0.192	0.198	0.194	0.194	0.194	0.205	0.198	0.210	0.185	0.187	0.196	0.203	0.216

**Table 4.** Estimates of evolutionary divergence between sequences. 16S: The number of base differences per site from between sequences are shown. The analysis involved 17 nucleotide sequences. All positions containing gaps and missing data were eliminated.

		There were a total of 392 positions in the final dataset.															
		1	2	3	4	5	6	7	8	9	10	11	12	13	14	15	16
**1**	*Scolopendra cingulata* (IZ-131446)																
**2**	*Cryptops anomalans* UK (IZ-131458)	0.390															
**3**	*C. anomalans* SW Serbia (1a)	0.372	0.092														
**4**	*C. anomalans* SW Serbia (1b)	0.372	0.092	0.000													
**5**	*C. anomalans* SW Serbia (7)	0.372	0.094	0.003	0.003												
**6**	*C. anomalans* southern Serbia (3)	0.372	0.082	0.036	0.036	0.038											
**7**	*C. anomalans* Belgrade, Serbia (8)	0.367	0.092	0.041	0.041	0.041	0.018										
**8**	*C. anomalans* Belgrade, Serbia (13)	0.372	0.089	0.043	0.043	0.043	0.020	0.008									
**9**	*C. anomalans* Belgrade, Serbia (9)	0.372	0.087	0.041	0.041	0.041	0.010	0.008	0.010								
**10**	*C. anomalans* southern Serbia (12)	0.365	0.084	0.033	0.033	0.036	0.018	0.031	0.033	0.023							
**11**	*C. anomalans* SE Romania (54a)	0.372	0.092	0.059	0.059	0.059	0.048	0.054	0.051	0.048	0.051						
**12**	*C. anomalans* Bucharest Romania (57a)	0.372	0.099	0.066	0.066	0.066	0.054	0.059	0.056	0.054	0.059	0.013					
**13**	*Cryptops speleorex* sp. nov. Movile cave, Romania (K4)	0.365	0.099	0.082	0.082	0.082	0.066	0.074	0.071	0.069	0.071	0.066	0.069				
**14**	*Cryptops speleorex* sp. nov. Movile cave, Romania (K3)	0.383	0.112	0.087	0.087	0.084	0.071	0.077	0.074	0.069	0.082	0.079	0.082	0.066			
**15**	*C. anomalans* SE Serbia cave (4)	0.385	0.084	0.094	0.094	0.097	0.077	0.087	0.084	0.082	0.079	0.097	0.107	0.107	0.125		
**16**	*C. parisi* UK (IZ-130592)	0.355	0.217	0.209	0.209	0.212	0.214	0.227	0.224	0.222	0.219	0.224	0.227	0.232	0.235	0.232	
**17**	*C. hortensis* UK (IZ-130582)	0.360	0.230	0.217	0.217	0.219	0.222	0.235	0.230	0.232	0.224	0.235	0.245	0.230	0.219	0.230	0.260

**Table 5.** Estimates of evolutionary divergence between sequences. 28S: The number of base differences per site from between sequences are shown. The analysis involved 18 nucleotide sequences.

		All positions containing gaps and missing data were eliminated. There were a total of 316 positions in the final dataset.																
		1	2	3	4	5	6	7	8	9	10	11	12	13	14	15	16	17
**1**	*Scolopendra cingulata* (IZ-131446)																	
**2**	*Cryptops anomalans* UK (IZ-131458)	0.187																
**3**	*C. anomalans* Bucharest Romania (57a)	0.187	0.000															
**4**	*C. anomalans* southern Serbia (2)	0.190	0.003	0.003														
**5**	*C. anomalans* southern Serbia (3)	0.190	0.003	0.003	0.000													
**6**	*C. anomalans* Belgrade, Serbia (8)	0.190	0.003	0.003	0.000	0.000												
**7**	*C. anomalans* Belgrade, Serbia (9)	0.190	0.003	0.003	0.000	0.000	0.000											
**8**	*C. anomalans* southern Serbia (12)	0.190	0.003	0.003	0.000	0.000	0.000	0.000										
**9**	*C. anomalans* Belgrade, Serbia (13)	0.190	0.003	0.003	0.000	0.000	0.000	0.000	0.000									
**10**	*Cryptops speleorex* sp. nov. Movile cave, Romania (K3)	0.190	0.003	0.003	0.000	0.000	0.000	0.000	0.000	0.000								
**11**	*Cryptops speleorex* sp. nov. Movile cave, Romania (K4)	0.190	0.003	0.003	0.000	0.000	0.000	0.000	0.000	0.000	0.000							
**12**	*C. anomalans* SE Romania (54a)	0.190	0.003	0.003	0.000	0.000	0.000	0.000	0.000	0.000	0.000	0.000						
**13**	*C. anomalans* SW Serbia (1a)	0.190	0.003	0.003	0.000	0.000	0.000	0.000	0.000	0.000	0.000	0.000	0.000					
**14**	*C. anomalans* SW Serbia (1b)	0.190	0.003	0.003	0.000	0.000	0.000	0.000	0.000	0.000	0.000	0.000	0.000	0.000				
**15**	*C. anomalans* SW Serbia (7)	0.190	0.003	0.003	0.000	0.000	0.000	0.000	0.000	0.000	0.000	0.000	0.000	0.000	0.000			
**16**	*C. anomalans* SE Serbia cave (4)	0.184	0.006	0.006	0.009	0.009	0.009	0.009	0.009	0.009	0.009	0.009	0.009	0.009	0.009	0.009		
**17**	*C. parisi* UK (IZ-130592)	0.196	0.054	0.054	0.057	0.057	0.057	0.057	0.057	0.057	0.057	0.057	0.057	0.057	0.057	0.057	0.057	
**18**	*C. hortensis* UK (IZ-130582)	0.190	0.063	0.063	0.066	0.066	0.066	0.066	0.066	0.066	0.066	0.066	0.066	0.066	0.066	0.066	0.070	0.079

### Key for identification of cave-specialized (troglomorphic/troglophilic) *Cryptops*

**Table:** 

1	Forcipular coxosternal margin with blunt, rounded or slightly flattened, hyaline lobes; tarsungulum very short	**C. (Paracryptops) indicus**
–	Forcipular coxosternal margin without hyaline lobes; tarsungulum moderate or long	**3**
3	Trigonal sutures present on the posterior part of sternites. Tarsus of most legs bipartite	**Cryptops (Trigonocryptops)** ^1^
–	Sternal trigonal sutures absent. Tarsus of most legs a single article	**Cryptops (Cryptops)**
5	Ultimate legs with saw teeth present from prefemur to tarsus 2, saw teeth formula: 28-30-14-17-17	***C. spelaeoraptor* Ázara & Ferreira, 2014**
–	Ultimate legs with saw teeth present on tibia and tarsus 1 only	**7**
7	T1 with transverse suture only	**9**
–	T1 with transfer and other sutures	**11**
9	Head without paramedian sutures; length: 19 mm, antennae short, 3+3 saw teeth on tibia and tarsus of ultimate legs	***C. beroni***
–	Head with incomplete paramedian sutures on the posterior half and the anteriormost quarter of the cephalic plate; length: 28–29 mm; antennae long, 4+9 saw teeth on tibia and tarsus 1 of ultimate leg	***C. illyricus***
11	T1 with inverted Y-shaped sutures	***C. legagus* Edgecombe, Akkari, Netherlands, Du Preez, 2020**
–	T1 with transverse and/or paramedian sutures	**13**
13	T1 with transverse suture and two paramedian sutures; prefemur and femur of ultimate legs with dorsodistal spinous process; small species, ca 15 mm, cave in India	***C. kempi***
–	T1 with transverse suture and U-shaped or cruciform suture; prefemur and femur of ultimate legs without dorsodistal spinous process; caves in Europe	**15**
15	T1 with transverse and cruciform sutures; head with 2 complete paramedian sutures, large species	***Cryptops speleorex* sp. nov.**
–	T1 with transverse suture and characteristic U-shaped suture attached to it; head with incomplete paramedian sutures	**17**
17	Labrum tridentate	**19**
–	Labrum unidentate	**21**
19	Antennae short, head plate with incomplete anterior and posterior paramedian sutures; saw teeth on tibia and tarsus in combination 13+6	***C. dianae***
–	Antennae long, head plate with posterior paramedian sutures only	***C. umbricus umbricus***
21	Head with two incomplete posterior paramedian sutures only; anterior margin of forcipular coxosternite strongly convex and covered by spiniform setae, cave in France	***C. umbricus lewisi***
–	Head with two incomplete short posterior paramedian sutures only; anterior margin of forcipular coxosternite slightly rounded and barely protuberant; spiniform setae missing, cave on Tenerife	***C. vulcanicus***

## Discussion

Scolopendromorphs are strictly terrestrial and most species are found in forest leaf litter, decomposed wood, under bark of dead trees, in the soil, under stones or in caves in the temperate and tropical areas of the world. Few species are well adapted to eremic environments (Minelli and Golovatch 2013), occasionally in atypical habitats such as forest canopy (Lewis 1982; Phillips et al. 2020) or tropical rivers (Siriwut et al. 2016). Although less common than lithobiomorphs, scolopendromorphs may occur in caves, where they are represented with some highly adapted species, mainly from the family Cryptopidae. Other families are only marginally recorded in caves: Scolopocryptopidae (genera *Thalkethops* Crabill, 1960 and *Newportia* Gervais, 1847 with several species from American caves, including several troglobites), Plutoniumidae (genera *Plutonium* Cavanna, 1881 and *Theatops* Newport, 1844) in European caves and Scolopendridae (genus *Otostigmus Porat*, 1876; *O. cooperi* Chamberlin, 1942 inhabits Chilibrilo caves in Panama (Chamberlin 1942); *Otostigmus troglodytes* Ribaut, 1914 found in a cave near Tanga, Tanzania (Ribaut 1914)). The genus *Cryptops* is by far the most frequent in the caves worldwide with some 18–20 species found in caves in South Europe (Spain, France, Italy, Greece), Canary Islands, Cuba, Brazil, Australia and Africa. Troglomorphic species are known from the nominate subgenus, and the subgenera *Trigonocryptops* and *Paracryptops* (see Table 6).

**Table 6.** An annotated list of the troglobitic/troglophilic *Cryptops* species in the world.

Species	Distribution	Category	References
Cryptops (Cryptops) beroni Matic & Stavropoulos, 1988	Greece: Crete, Acrotiri, Cave Katholiko	Troglobite?	Matic and Stavropoulos (1988)
Cryptops (Trigonocryptops) camoowealensis Edgecombe, 2006	Australia: Queensland, Camooweal area, Five O’Clock Cave	Troglobite	Edgecombe (2006)
Cryptops (Trigonocryptops) cavernicolus Negrea & Fundora Martinez, 1977	Cuba	Troglobite	Matic et al. (1977)
Cryptops (Cryptops) dianae Matic & Stavropoulos, 1990	Greece: Thassos Island, cave Dracotrypa	unknown	Matic and Stavropoulos (1990)
Cryptops (Trigonocryptops) hephaestus Ázara & Ferreira, 2013	Brazil: known from three iron ore caves of the “Quadrilátero Ferrífero” (Iron Quadrangle) in Minas Gerais in Mariana and Itabirito municipalities	Troglophile	Ázara and Ferreira (2013), Chagas-Jr and Bichuette (2018)
Cryptops (Cryptops) illyricus Verhoeff, 1933	Caves only?; Slovenia and Croatia		Verhoeff 1933
Cryptops (Trigonocryptops) iporangensis Ázara & Ferreira, 2013	Brazil: known from four caves (Ressurgência das Areias de Água Quente, Gruta Monjolinho, Caverna Alambari de Baixo, Caverna Santana) in Iporanga, São Paulo	Troglobite	Ázara and Ferreira (2013), Chagas-Jr and Bichuette (2018)
Cryptops (Paracryptops) indicus (Silvestri, 1924)	India: Assam, Garo Hills, Siju Cave	Troglophile	(Silvestri 1924)
Cryptops (Cryptops) kempi Silvestri, 1924	India: Assam, Garo Hills, Siju Cave	Troglophile	(Silvestri 1924)
Cryptops (Cryptops) legagus Edgecombe, Akkari, Netherlands, Du Preez, 2020	Botswana: Diviner’s Cave (Koanaka Hills) and Dimapo Cave (Gcwihaba Hills)	Epigean/Troglophile?	Edgecombe et al. (2020)
Cryptops (Trigonocryptops) longicornis (Ribaut, 1915)	Caves in Spain	Troglobite	Ribaut (1915)
Cryptops (Cryptops) speleorex sp. nov.	Romania: Mangalia, Movile Cave	Troglobite	This paper (see also Negrea 1993)
Cryptops (Trigonocryptops) roeplainsensis Edgecombe, 2005	Australia: known from three caves (Nurina Cave 6N-46, Burnabbie Cave, cave 6N-1327), Roe Plains	Troglobite	Edgecombe (2005)
Cryptops (Cryptops) spelaeoraptor Ázara & Ferreira, 2014	Brazil: Bahia, Campo Formoso, only known from the type locality, Toca do Gonçalo Cave		Ázara and Ferreira (2014), Chagas-Jr and Bichuette (2018).
Cryptops (Trigonocryptops) troglobius Matic, Negrea & Fundora Martinez, 1977	Cuba	Troglobite	Matic et al. (1977)
Cryptops (Cryptops) umbricus umbricus Verhoeff, 1931	Caves in France and Italy but also found outside caves	Troglophile	Verhoeff (1931), Matic (1960), Iorio and Minelli (2005), Iorio and Geoffroy (2007, 2008), Iorio (2010)
Syn. *Cryptops jeanneli* Matic, 1960			
*Cryptops umbricus ischianus* Verhoeff, 1942			
Cryptops (Cryptops) umbricus lewisi Iorio, 2010	France: Alpes-Maritimes, Gourdon, Aven du Fourchu Cave	Troglobite	Iorio (2010)
Cryptops (Cryptops) vulcanicus Zapparoli, 1990	Spain: Tenerife Island, Cueva Felipe Reventón	Troglobite	Zapparoli (1990)

Several morphological characters traditionally used in centipedes taxonomy could be subject to intraspecific variation related to postembryonic development, animal life stage and ecology (Akkari et al. 2017). This might render species identification problematic in some cases and generates taxonomic errors. This is also true for such a highly variable and widely distributed species as *C. anomalans*. In fact, nine species and subspecies were hitherto synonymised with this species (see Krapelin 1903; Verhoeff 1931; Crabill 1962; Zapparoli 2002). Three subspecies are still listed as valid for it (Chilobase 2.0). Now the identity of these taxa and the presence of any possible cryptic species within *C. anomalans* could only be revealed via an integrative study combining morphological and molecular markers. Whereas clear molecular differences are here indicated by the different markers and the high interspecific distance between *C. anomalans* and the newly described species *C. speleorex* sp. nov., the morphological comparison was not as straightforward since both species show several similarities, including an overlapping in size. While several of the differences observed between both species (Table 2) could be understood as a clear indication of troglomorphism in *C. speleorex* sp. nov. such as the elongation of appendages, a few other characters including the number of saw teeth on tibia and tarsus 1 of the ultimate legs, number of coxal pores and the shape of spiracles were diagnostic to separate both species.

Intraspecific distance between the two sequenced *Cryptops speleorex* sp. nov. specimens is relatively high in comparison to the detected interspecific variation (Tables 3–5) raising a question whether these two specimens could actually be interpreted as two separate species. However, this variation is only shown in the two mitochondrial markers – there are no morphological differences (or any difference in their nuclear 28S marker) between the *C. speleorex* sp. nov. specimens. As Morgan-Richards et al. (2017) well explains, cryptic speciation should never be used as a null hypothesis in the absence of phenotypic or nuclear data supporting it. Instead, “the origin of the divergent mtDNA haplogroups might result from complex biogeographical scenarios or they might simply represent normal, stochastic processes of mutation and extinction of a non-recombining locus within a large population”.

### Taxonomic and evolutionary implications of *C. speleorex* sp. nov.

**Figure 9.**
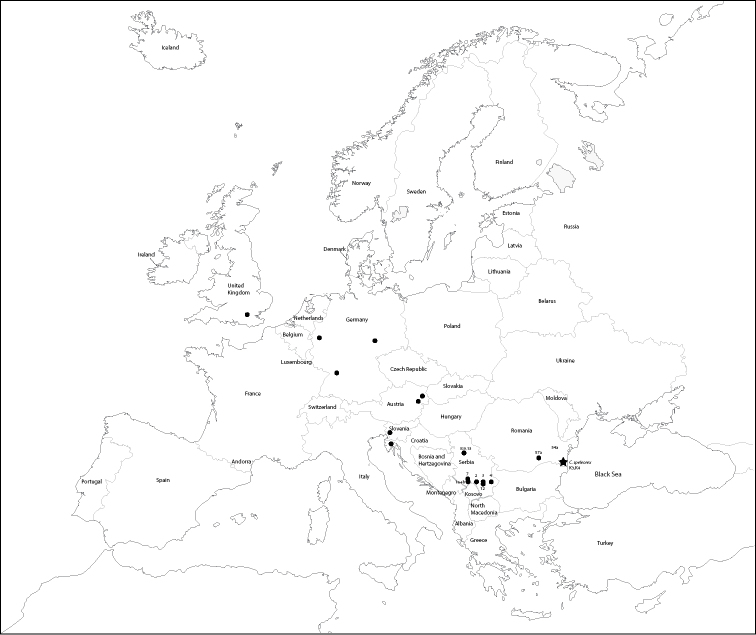
Map of Europe showing geographic distribution of *Cryptops* specimens analyzed herein. Asterisk – *C. speleorex* sp. nov. Dot – other *Cryptops* spp. used in the study (see Table 1 for details).

The type locality of *C. anomalans* is unknown and therefore it is impossible to conclude which part (if any) of the studied population is the actual *C. anomalans* described by Newport (1844). Before this study, only a handful of *C. anomalans* specimens from a limited geographic range had been sequenced (Spelda et al. 2011; Vahtera et al. 2013; Wesener et al. 2016). We acknowledge that describing *C. speleorex* sp. nov. as a new species leaves *C. anomalans* paraphyletic and that monophyly is violated by this taxonomic act. However, we view this as an inevitable consequence of speciation with a particular evolutionary implication, i.e., that *C. speleorex* sp. nov. evolved within what is currently known as *C. anomalans*. It is worth noting that the closest evolutionary relatives of *C. speleorex* sp. nov. appear to be the *C. anomalans* specimens from Serbia (excluding the sample number 4) and Romania (Figs 8, 9). This means that they are most closely related to each other than either of them is to the rest of the studied *C. anomalans* populations. The current situation with *C. anomalans* should not be seen as a failed taxonomy but as a natural consequence when new data from a widespread species is obtained.

## Supplementary Material

XML Treatment for Cryptops (Cryptops) anomalans

XML Treatment for Cryptops (Cryptops) speleorex

## Footnotes

Here belong: C. camoowealensis, C. cavernicolus, C. hephaestus, C. iporangensis, C. longicornis, C. roeplainsensis, C. troglobius
